# Recurrent Superior Vena Cava Syndrome in a Patient with Sarcoidosis and Pancreatic Adenocarcinoma: A Case Report and Literature Review

**DOI:** 10.3390/medicines7090056

**Published:** 2020-09-04

**Authors:** Ganesh Shenoy, Yunsung Kim, Kyra Newmaster, Kathryn L. McGillen, Francesca Ruggiero, Nelson S. Yee

**Affiliations:** 1 Penn State College of Medicine Medical Scientist Training Program, Hershey, PA 17033, USA; gshenoy@pennstatehealth.psu.edu (G.S.); ykim3@pennstatehealth.psu.edu (Y.K.); knewmaster@pennstatehealth.psu.edu (K.N.); 2 Department of Radiology, Penn State Health Milton S. Hershey Medical Center, Hershey, PA 17033, USA; kmcgillen@pennstatehealth.psu.edu; 3 Department of Pathology, Penn State Health Milton S. Hershey Medical Center, Hershey, PA 17033, USA; fruggiero@pennstatehealth.psu.edu; 4 Division of Hematology-Oncology, Department of Medicine, Penn State Health Milton S. Hershey Medical Center, Next-Generation Therapies Program, Penn State Cancer Institute, The Pennsylvania State University College of Medicine, Hershey, PA 17033, USA

**Keywords:** pancreatic adenocarcinoma, pancreatic cancer, sarcoidosis, superior vena cava syndrome, venous thrombosis

## Abstract

**Background:** Superior vena cava (SVC) syndrome may result from extravascular compression or intravascular obstruction such as thrombosis. Recurrent venous thrombosis is typically associated with a hypercoagulable state such as malignancy, and inheritable or acquired coagulopathy. Sarcoidosis is a derangement of the immune system, and it has been associated with malignant diseases and hypercoagulation. The association of pancreatic cancer and sarcoidosis with SVC syndrome has not been reported previously. Here, we present a case of recurrent venous thrombosis causing SVC syndrome in a patient with pancreatic ductal adenocarcinoma and underlying thoracic sarcoidosis. **Methods:** The patient’s electronic health record was retrospectively analyzed. **Results:** A 66-year-old woman with pancreatic adenocarcinoma was treated with neoadjuvant chemotherapy followed by Whipple procedure, before developing tumor recurrence in the liver. Her treatment course was complicated with repeated incidents of venous thrombosis in the presence of a central venous catheter leading to recurrent SVC syndrome, which resolved with anti-coagulation. **Conclusions:** This case raises a plausible inter-relationship between sarcoidosis, pancreatic cancer, and hypercoagulable state. We suggest that patients with multiple risk factors for developing venous thrombosis should be carefully monitored for any thrombotic event, and they may benefit from prophylactic anti-coagulation.

## 1. Introduction

Superior vena cava (SVC) syndrome is a medical emergency that comprises a group of symptoms caused by obstruction of the SVC. Characteristic symptoms of SVC syndrome include edema of the face, neck, upper-body and extremities that are often accompanied by dyspnea. The buildup of pressure proximal to the SVC obstruction results in the distension of veins on the neck and chest and dilation of collateral vessels that return blood to the heart [1,2]. An estimated 60 to 85% of cases of SVC syndrome are thought to be due to malignancy, with lung cancer and non-Hodgkin lymphoma being the most commonly cited cancers associated with the syndrome [2]. As a result of their location, cancers of the lungs and mediastinum can compress the SVC from mass effect or invade into the lumen of the SVC resulting in its obstruction. Several cases of SVC syndrome have been reported as a rare manifestation of sarcoidosis, in which the thoracic granuloma causes compression of the SVC [3,4,5,6,7].

The other cases of SVC syndrome, particularly in acute settings, have been attributed to thrombosis, resulting in intraluminal obstruction of the SVC. Thrombotic etiology of SVC syndrome has been recently reported to be on the rise due to an increased usage of intravascular devices, which are known to increase the risk of thrombotic events [1,2]. Spontaneous thrombosis of the SVC has also been reported in patients with hypercoagulable states or malignancies as a paraneoplastic manifestation [8,9,10,11,12]. While sarcoidosis has been associated with hypercoagulation [13], SVC syndrome caused by venous thrombosis in the setting of sarcoidosis, cancer, and a venous catheter has not been reported previously.

Here, we present the case of a patient with a history of thoracic sarcoidosis, who developed pancreatic adenocarcinoma and whose treatment course with systemic chemotherapy was complicated with multiple thrombotic events leading to SVC syndrome. This represents the first reported case in which recurrent SVC syndrome caused by venous thrombosis may be attributed to multiple predisposing factors that concurrently exist in the individual patient.

## 2. Materials and Methods

This is a retrospective review of the electronic health record, images, and histopathology at our institution. We have obtained the written informed consent of the patient’s spouse since the patient is deceased. The Human Subjects Protection Office of the Penn State Health Milton S. Hershey Medical Center determined that this case report does not meet the definition of human subject research as defined in 45 CFR 46.102 (d) and/or (f); Institutional Review Board (IRB) review and approval is not required. Review of the literature on the associations among sarcoidosis, malignancy, hypercoagulability, and SVC syndrome was conducted.

## 3. Case Presentation

A 66-year-old Caucasian woman presented with acholic stool and dark, tea-colored urine accompanied with a feeling of discomfort in the posterior thoracic region. She reported an unintentional weight loss of 15 pounds over 5 months. Her past medical history included thoracic sarcoidosis previously treated with steroid medication by her primary care physician, non-insulin dependent diabetes mellitus, hyperlipidemia, hypertension, gastroesophageal reflux disease, and allergic rhinitis. She denied any history of smoking cigarettes or consuming alcohol. Her family history was notable for a brother with lung cancer and prior asbestos exposure; otherwise, she did not have any known family history of pancreatic cancer, sarcoidosis, or thrombotic disorder. Her home medications included azelastine spray pump, diclofenac sodium, glipizide, lisinopril, loratadine, magnesium oxide, metformin, omeprazole, and simvastatin.

Upon physical examination, the patient appeared in no acute distress and had a pleasant mood. Blood pressure was 188/93, heart rate 105 beats per minutes. Sclera were slightly icteric, and skin mildly jaundiced. Lungs were clear to auscultation. A soft systolic murmur was appreciated in her right upper chest. Bowel sounds were normal, the abdomen was soft and non-tender, and no hepatosplenomegaly or mass was palpated. There was no peripheral edema, with an unremarkable remaining physical examination.

**Figure 1:**
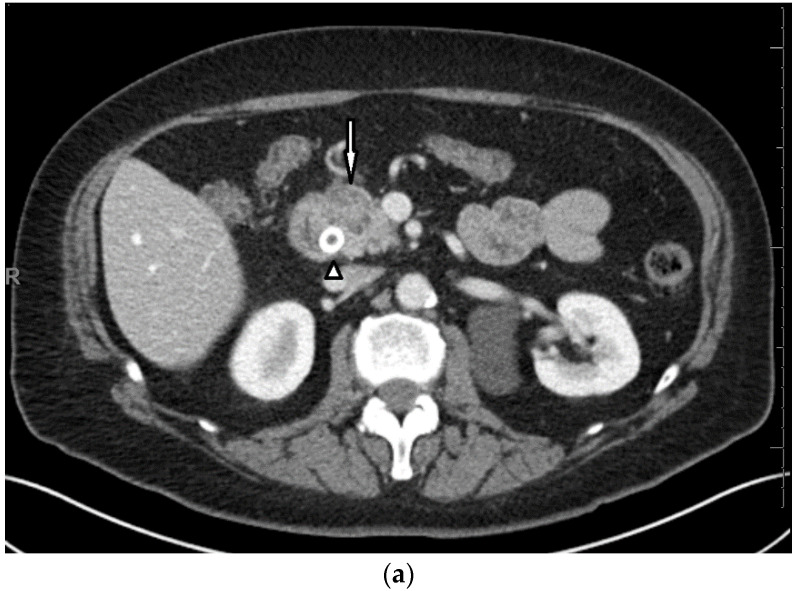

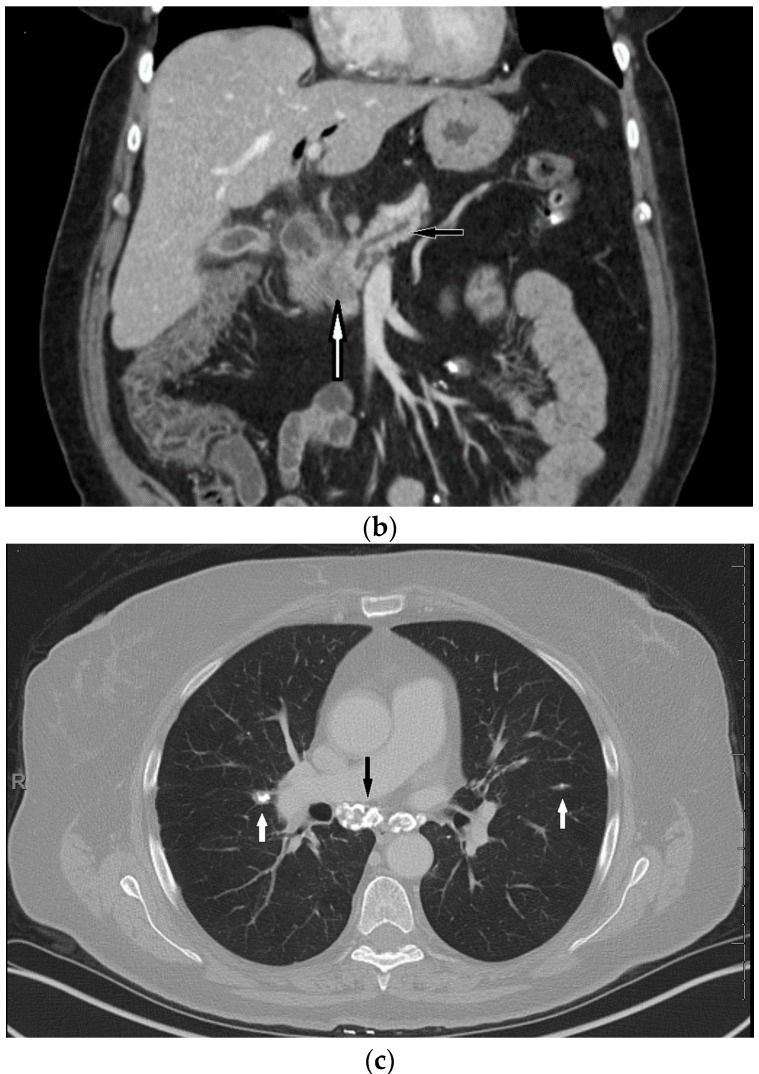
Axial (**a**) and coronal (**b**) intravenous contrast-enhanced CT scans of the chest, abdomen, and pelvis at initial presentation show a 3.1 × 2.1 cm hypo-enhancing mass in the head of the pancreas (white arrows) with mild upstream pancreatic ductal dilatation and parenchymal atrophy (black arrow). The dilated biliary tree has been decompressed via a metal common bile duct stent (white arrow head); (**c**) Images through the chest on lung windows show calcified mediastinal lymph nodes (black arrow) and calcified pulmonary nodules (white arrows)—non-specific sequela of prior granulomatous process, including sarcoid.

The laboratory results were notable for elevated total bilirubin 3.2 mg/dL (normal 0–1.2 mg/dL), aspartate aminotransferase 278 U/L (normal 0–32 U/L), alanine aminotransferase 320 U/L (normal 0–33 U/L, and alkaline phosphatase 424 U/L (normal 35–115 U/L). Glucose was found to be elevated at 307 mg/dL (normal 70–100 mg/dL). An intravenous (IV) contrast-enhanced CT scan of the chest, abdomen and pelvis showed a mass in the pancreatic head. In the chest, calcified nodules in the lungs and calcified mediastinal lymph nodes were present, representing non-specific sequela of a prior granulomatous process and consistent with the known history of sarcoidosis (Figure 1). Endoscopic retrograde cholangiopancreatography (ERCP) was performed for the pancreatic mass, and it showed a high-grade, malignant-appearing stricture. She underwent a biliary sphincterotomy and placement of a metal biliary stent in the common bile duct. Endoscopic ultrasonography showed a hypoechoic mass measuring 29.5 × 24.5 mm in the pancreatic head with invasion into the portal vein and proximal portion of the duodenum along with peri-pancreatic lymphadenopathy. Fine-needle aspiration of the pancreatic mass revealed malignant cells consistent with pancreatic ductal adenocarcinoma.

Considering her uncontrolled diabetes mellitus, high serum level of CA 19-9 (4511 U/mL; normal 0–36 U/mL), and vascular involvement by the pancreatic mass, it was decided that the patient would receive neoadjuvant chemotherapy and then be re-evaluated for surgical resection of tumor. A double-lumen MediPort was placed in the right internal jugular vein, with the catheter ending at the junction between SVC and right atrium. She started to receive neoadjuvant chemotherapy with oxaliplatin, irinotecan, leucovorin, and 5-fluorouracil (FOLFIRINOX).

One week following the initiation of the first cycle of chemotherapy, the patient presented with a non-neutropenic fever (102 °F). Diagnostic evaluation with labs, cultures, urinalysis, and a chest x-ray was unremarkable, and the fever was resolved with acetaminophen. However, approximately two weeks later, she again presented to the Emergency Department (ED) with non-neutropenic fever (102.2 °F). Blood cultures grew Klebsiella pneumoniae, and the urine culture was positive for >100,000 colonies of Group B streptococci. The patient was treated with a course of piperacillin and tazobactam and subsequently discharged with levofloxacin and metronidazole.

**Figure 2:**
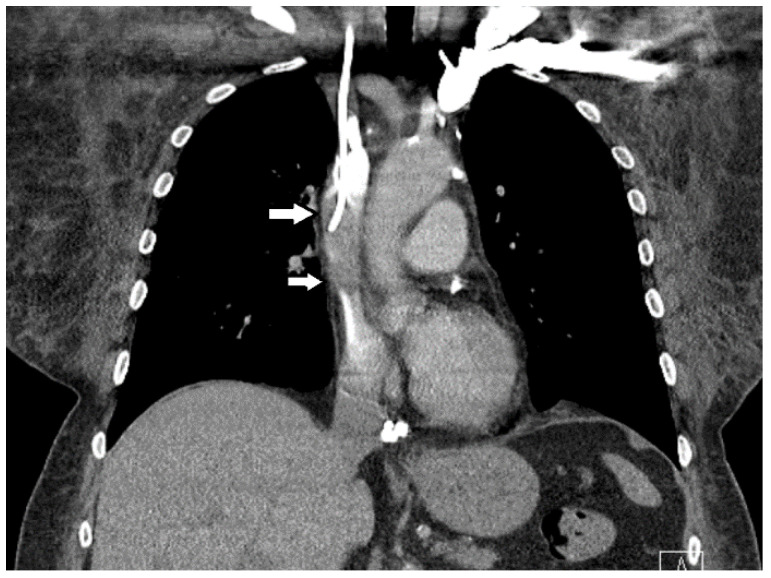
An IV contrast-enhanced CT scan of the chest timed to evaluate the superior vena cava shows a thrombus along the port catheter (white arrows) involving up to half of the lumen, shown on coronal reconstruction. There is a small amount of the thrombus within the right atrium (not shown).

**Figure 3:**
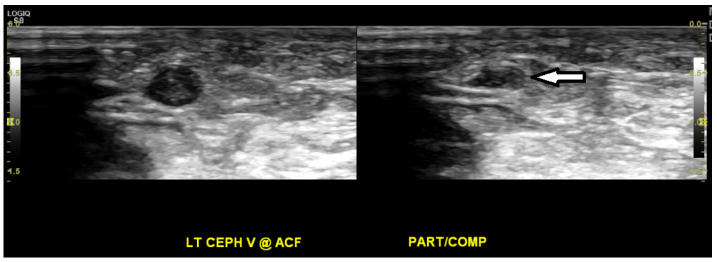
Ultrasound venous duplex imaging of the bilateral upper extremities shows echogenic, non-compressible material in the left cephalic vein, representing acute, non-occlusive thrombosis (white arrow), and also in the right cephalic vein in the distal arm (not shown).

Approximately three months after starting chemotherapy, she presented to the ED with new symptoms of dyspnea, facial edema, flushing of the neck, and fatigue. Physical examination was remarkable for peri-orbital and facial edema, plethora of the upper chest and neck, distention of superficial veins in the neck, and edema in the bilateral upper extremities. An IV contrast-enhanced CT scan of the chest showed a thrombus around the port catheter involving the SVC (Figure 2), while a CT scan of the neck showed no thrombosis in the internal jugular veins. Venous duplex imaging showed acute, non-occlusive thrombosis in the bilateral cephalic veins (Figure 3). These physical and radiological findings were consistent with SVC syndrome. Blood tests for hypercoagulation were not conducted. Vascular surgery was consulted and determined that no acute surgery was required as the thrombus was non-occlusive. The patient was initially anti-coagulated using IV bolus and infusion of heparin to achieve therapeutic activated partial thromboplastin time (aPTT, 60–80 s). As her edema improved, she was subsequently treated with enoxaparin (1 mg/kg) 80 mg subcutaneously every 12 h and discharged with instructions to continue this anti-coagulation regimen.

Twenty-three days later, the patient again presented to the ED, now with abdominal pain and non-bloody emesis. She was found to have a fever (102 °F) and hypotension, the latter of which improved with 2 L of normal saline. An IV contrast-enhanced CT scan of the abdomen showed fat stranding near the common bile duct stent and features of cholangitis. Blood cultures were positive for Enterobacter cloacae. The patient was treated with cefepime, metronidazole, and vancomycin and enoxaparin was temporarily discontinued in preparation for endoscopy. ERCP showed worsening biliary tract obstruction caused by a mass in the lower third of the main duct, and a new biliary stent was placed in the common bile duct. Transthoracic echocardiogram showed no evidence of endocarditis. Surveillance blood cultures were negative, and she was discharged to home with levofloxacin, metronidazole, and instructions to continue enoxaparin 80 mg subcutaneously every 12 h for anti-coagulation.

**Figure 4:**
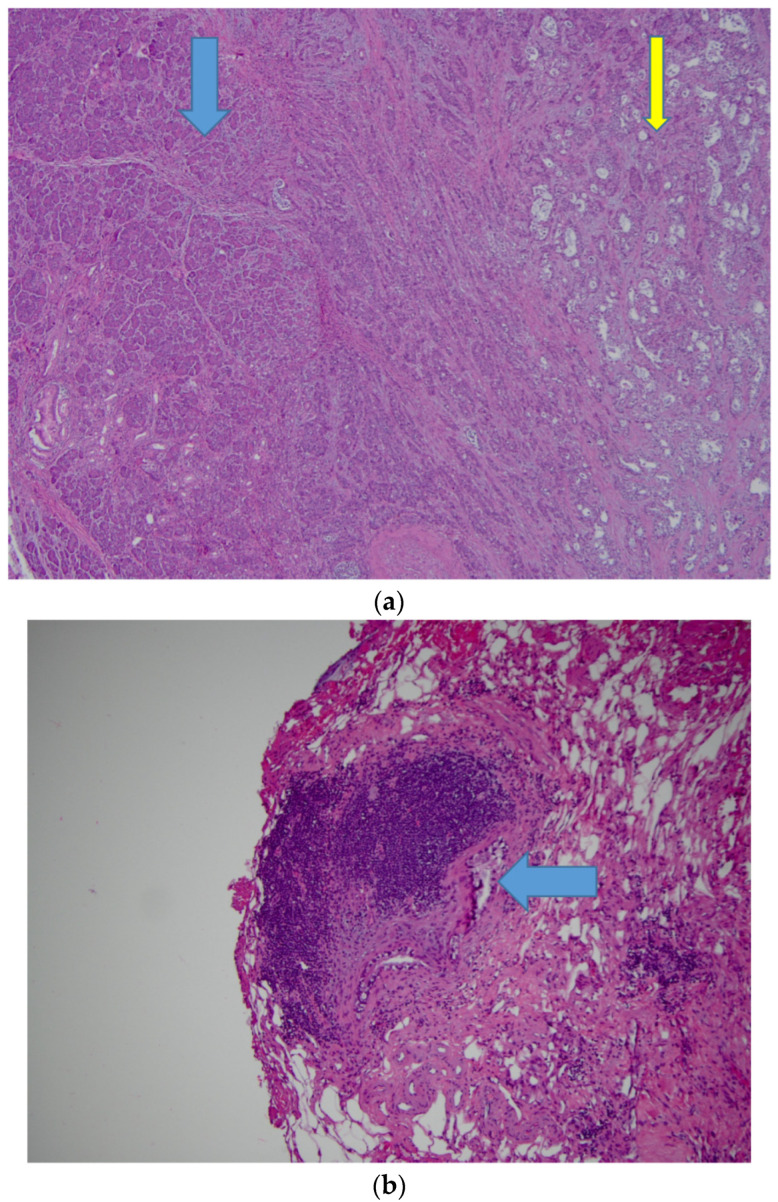
Pancreaticoduodenectomy (Whipple resection) and partial pancreatectomy. (**a**) Pancreas, 40×, hematoxylin and eosin (H&E) stain. The blue arrow indicates normal pancreatic parenchyma; the yellow arrow indicates moderately differentiated adenocarcinoma, characterized by infiltrating glands. (**b**) Lymph node, 100×, H&E stain. Lymph node with metastatic adenocarcinoma (blue arrow indicates metastasis).

Upon completion of five cycles of FOLFIRINOX, the CA 19-9 level had reduced to 342.2 U/mL. Restaging CT scans with IV contrast showed that the known primary pancreatic lesion and the previously identified thrombus around the port catheter involving the SVC were no longer visualized. Treatment with enoxaparin was discontinued and the patient underwent pancreaticoduodenectomy (Whipple resection). Pathology of the surgical specimen revealed ductal adenocarcinoma in the pancreatic head and two lymph nodes (Figure 4). A double-lumen peripherally inserted central catheter (PICC) was placed in the left basilic vein with the final tip position in the SVC for total parenteral nutrition due to oral intolerance of diet. Three weeks later, she presented to the ED with edema and pain in the right upper extremity. Venous duplex ultrasound imaging of the right upper extremity did not reveal any deep or superficial vein thrombus.

**Figure 5:**
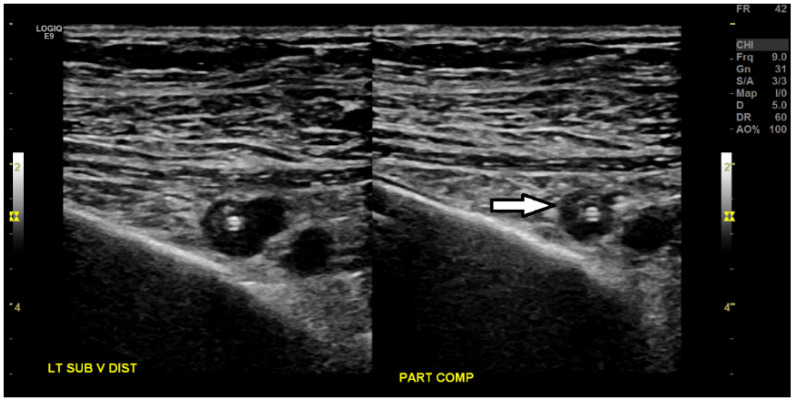
Ultrasound venous duplex imaging of the bilateral upper extremities shows an acute, partially occlusive deep-vein thrombus within the left subclavian vein along an indwelling venous catheter (white arrow).

**Figure 6:**
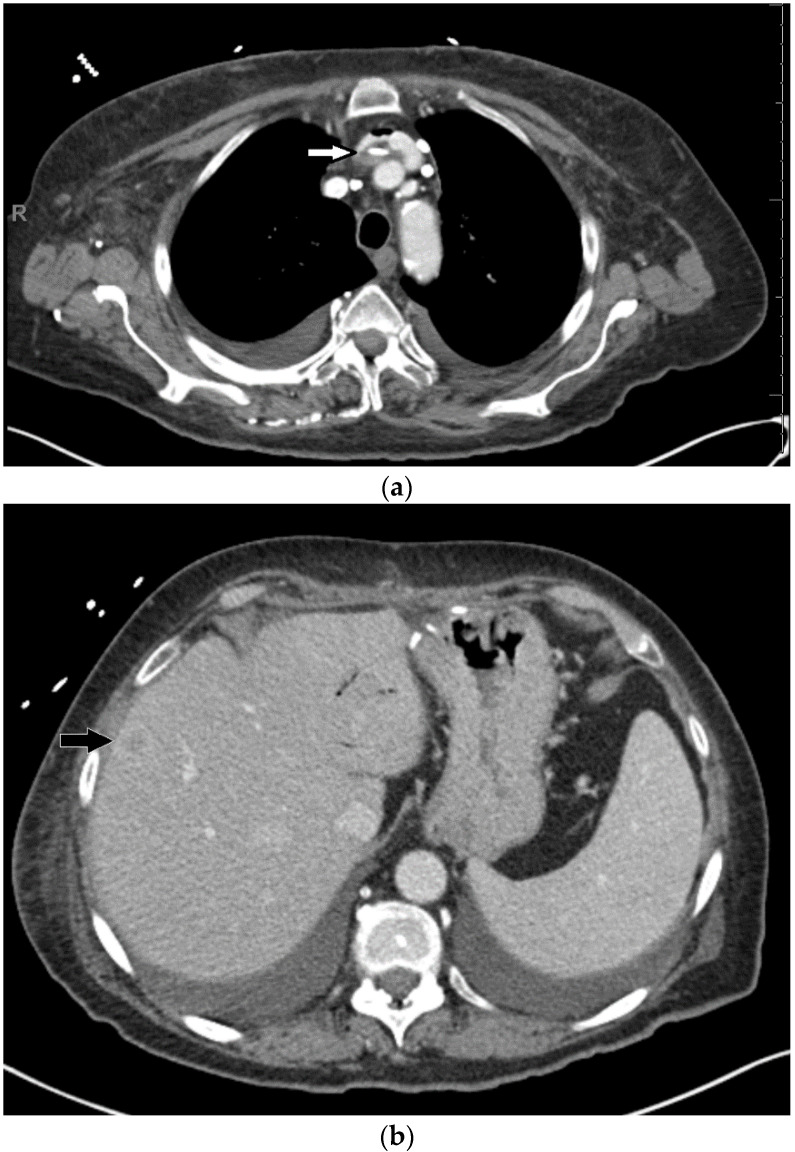
IV contrast-enhanced CT scan of the neck, chest, abdomen, and pelvis shows (**a**) a left-sided catheter with surrounding hypodense thrombus in the left brachiocephalic vein (white arrow), and small thrombus in the lower superior vena cava (SVC) at the SVC/atrial junction (not shown). Trace amount of iatrogenic air is also present immediately anterior to the clot in the brachiocephalic vein. (**b**) New hypo-enhancing liver lesions are also present (the black arrow denotes one of them).

One month following Whipple resection, the patient started receiving adjuvant chemotherapy using gemcitabine and capecitabine. Two and a half weeks later, she presented to the ED with peri-orbital edema, a distended superficial vein on the left side of the neck, as well as edema in the face, neck, and bilateral upper extremities. The serum levels of both D-dimer (2.56 µg/mL; normal < 0.54 µg/mL) and fibrinogen (443 mg/dL; normal 208–435 µg/mL) were elevated, and these findings are consistent with a thrombotic state. Venous duplex ultrasound imaging showed acute, partially occlusive thrombus in the left subclavian vein and the left basilic vein surrounding an indwelling venous catheter (Figure 5). A CT scan of the neck, chest, abdomen, and pelvis with IV contrast showed a thrombus along the left-sided PICC in the left brachiocephalic vein, a small thrombus in the lower SVC, and new hypodense lesions in the liver (Figure 6). The physical examination and radiological findings were consistent with recurrent SVC syndrome, the PICC was subsequently removed, and the patient was treated with IV bolus and infusion of heparin. Upon improvement of the edema, her anti-coagulation was converted to enoxaparin (1 mg/kg) 70 mg subcutaneously every 12 h with instructions to continue this regimen for prophylaxis.

CT-guided biopsy of the hepatic lesion revealed malignant cells consistent with metastatic pancreatic ductal adenocarcinoma. Molecular profiling of the biopsied liver lesion was notable for a pathogenic mutation G12V in exon 2 of the *KRAS* gene (by Caris Life Sciences^™^). Furthermore, the CA 19-9 level began to trend upward (1256 U/mL). Considering disease progression with tumor metastasis, the patient started to receive palliative chemotherapy using nanoparticle albumin-bound (nab) paclitaxel and gemcitabine. Following three 28-day cycles of nab-paclitaxel and gemcitabine, an IV contrast-enhanced CT scan showed a decrease in the size of some of the hepatic lesions, but also the presence of new lesions. Additionally, CA 19-9 levels were trending upward, suggesting tumor progression, and nab-paclitaxel and gemcitabine were discontinued.

**Figure 7:**
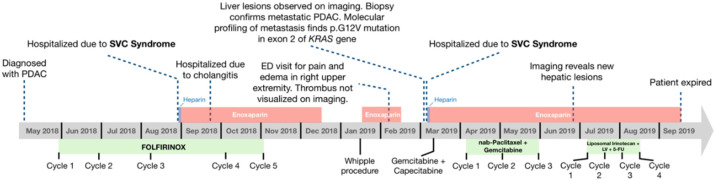
Timeline of major events during the course of the patient’s treatment for pancreatic ductal adenocarcinoma (PDAC). Complications and notable findings on imaging are listed above the timeline, while the patient’s chemotherapy regimens are indicated below.

A palliative chemotherapeutic regimen consisting of liposomal irinotecan, leucovorin, and 5-fluorouracil was initiated. Following four 14-day cycles, CA 19-9 levels (>9000 U/mL) continued to rise, suggesting continued tumor progression. Chemotherapy was discontinued and the patient opted to pursue hospice care. She expired 18 days after discontinuation of chemotherapy. The timeline of the clinical events is illustrated in Figure 7.

## 4. Discussion

In this case presentation, a patient with a history of thoracic sarcoidosis was diagnosed with pancreatic adenocarcinoma, who developed recurrent venous thrombosis causing SVC syndrome in the presence of indwelling venous catheters. Previous studies have suggested that sarcoidosis is associated with a hypercoagulable state and malignant diseases, including pancreatic adenocarcinoma [14,15,16,17]. This case report adds to the rare association of pancreatic adenocarcinoma with sarcoidosis. Moreover, to our knowledge, this represents the first reported case of SVC syndrome caused by venous thrombosis as a paraneoplastic manifestation of pancreatic cancer (Table 1). Since this patient has a diagnosis of pancreatic adenocarcinoma, which is known to be associated with a thrombotic state, a diagnostic evaluation for thrombophilia is not indicated.

While patients with cancer have increased thrombotic risk [18], most do not develop repeated and significantly symptomatic clots even in the presence of chronic indwelling lines. In agreement with this, the locations of multiple episodes of thrombosis in this patient do not always correlate with those of the venous catheters. The occurrence of repeated episodes of SVC syndrome in this patient can be related to the presence of multiple known risk factors of venous thrombosis. These include her underlying sarcoidosis, pancreatic adenocarcinoma, and systemic cytotoxic chemotherapy. While the exact pathogenic mechanism of sarcoidosis is unclear, this case report may help shed new light into the inter-relationship of sarcoidosis, pancreatic cancer, and venous thrombosis.

Numerous studies have examined the link between sarcoidosis and the development of cancers [13,19,20,21,22]. A systematic meta-analysis of 16 observational studies found that the relative-risk of sarcoidosis patients developing any cancer was 1.19 (95% CI 1.07–1.32). The most common cancer sites were the skin, followed by hematopoietic organs, upper-digestive organs, kidney, liver, and colorectum [23]. The results of a cohort study by Søgaard, et al. indicated that patients with sarcoidosis have a 20% increased risk of being diagnosed with cancer 3 to 10 years following the initial diagnosis of sarcoidosis [24]. These studies suggest that sarcoidosis is indeed a risk factor for the development of cancer. An incidental diagnosis of pancreatic cancer in this reported patient with underlying sarcoidosis cannot be excluded. Further evidence will be needed to demonstrate or support a relationship between pancreatic cancer and sarcoidosis. Whether the aberrant immune system or other pathogenic factors of sarcoidosis contribute to its association with cancer, including pancreatic cancer, remains to be determined.

Venous thrombosis and SVC syndrome have also been reported as rare manifestations of sarcoidosis. SVC syndrome has been shown to result from the direct compression of SVC by sarcoidosis-associated granuloma or lymphadenopathy [4,5,7,25]. While the exact mechanism behind the increased risk of thrombosis in patients with sarcoidosis is unknown, a few mechanisms have been proposed [26]. Firstly, the local inflammatory profile of sarcoid lesions is hypothesized to alter factors associated with coagulability [27]. A correlation between the location of sarcoid lesions and the location of thrombus formation has been reported [28]. These anatomic relationships suggest that local inflammation produced by sarcoid lesions modifies the surrounding environment into a hypercoagulable state [26]. Secondly, sarcoid lesions may alter the surrounding hemodynamics, predisposing to clot formation. Disruption in laminar flow is a well-characterized risk factor for thrombus formation [29]. Lastly, patients with sarcoidosis may suffer from complications associated with chronic steroid therapy (osteoporosis, obesity, pulmonary hypertension, etc.) that result in impaired mobility [30]—a known risk factor for the development of deep-vein thrombosis.

## 5. Conclusions

In this case report, a patient with underlying thoracic sarcoidosis was diagnosed with pancreatic adenocarcinoma; she received cytotoxic systemic chemotherapy and developed recurrent venous thrombosis causing SVC syndrome in the presence of an indwelling venous catheter. This study provides further support for an inter-relationship among sarcoidosis, hypercoagulation, and cancer. Caution about venous thrombosis should be raised in cancer patients with additional risk factors such as sarcoidosis, and prophylactic anti-coagulation for those individuals may be warranted.

## Figures and Tables

**Table 1:** SVC syndrome caused by intravascular thrombosis in association with malignancy.

Malignant disease	Age	Sex	Clinical Presentation of SVC Syndrome	Diagnosis of SVC Syndrome	Management	Reference
Pancreatic Adenocarcinoma	64	Female	Dyspnea, facial edema, flushing of neck, fatigue	Physical exam and IV contrast enhanced CT	IV heparin followed by enoxaparin	This Report
Soft Tissue Sarcoma	46	Female	Dyspnea, right arm pain, dysphagia	Spiral chest CT with contrast	Enoxaparin	[8]
Bronchogenic Carcinoma	55	Male	Dyspnea, edema of face, neck, and chest	Physical exam and Doppler ultrasonography	Low molecular weight heparin	[9]
Prostate Carcinoma	60	Male	Facial edema	Physical exam, contrast enhanced CT and MRI	Warfarin	[10]
Renal Cell Carcinoma	54	Male	Edema of face and neck	CT and MRI	IV Heparin	[11]
Ovarian Papillary Carcinoma	62	Female	Telangiectasias on face and anterior trunk	Thoracic CT	Acenocoumarol	[12]
